# Traumatic Brain Injury and NADPH Oxidase: A Deep Relationship

**DOI:** 10.1155/2015/370312

**Published:** 2015-03-31

**Authors:** Cristina Angeloni, Cecilia Prata, Francesco Vieceli Dalla Sega, Roberto Piperno, Silvana Hrelia

**Affiliations:** ^1^Department for Life Quality Studies, Alma Mater Studiorum-University of Bologna, C.so Augusto 237, 47921 Rimini, Italy; ^2^Department of Pharmacy and Biotechnology, Alma Mater Studiorum-University of Bologna, Via Irnerio 48, 40126 Bologna, Italy; ^3^Neurorehabilitation Unit, Emergency Department, AUSL of Bologna, Via B. Nigrisoli 2, 40133 Bologna, Italy

## Abstract

Traumatic brain injury (TBI) represents one of the major causes of mortality and disability in the world. TBI is characterized by primary damage resulting from the mechanical forces applied to the head as a direct result of the trauma and by the subsequent secondary injury due to a complex cascade of biochemical events that eventually lead to neuronal cell death. Oxidative stress plays a pivotal role in the genesis of the delayed harmful effects contributing to permanent damage. NADPH oxidases (Nox), ubiquitary membrane multisubunit enzymes whose unique function is the production of reactive oxygen species (ROS), have been shown to be a major source of ROS in the brain and to be involved in several neurological diseases. Emerging evidence demonstrates that Nox is upregulated after TBI, suggesting Nox critical role in the onset and development of this pathology. In this review, we summarize the current evidence about the role of Nox enzymes in the pathophysiology of TBI.

## 1. Introduction 

Traumatic brain injury (TBI) has become the leading cause of disability among young individuals and working age adults [1]. World Health Organization (WHO) has predicted that TBI will be the third leading cause of global mortality and disability by 2020 [2]. In the European Union over a million hospital admissions per year are due to TBI [3], making it one of the major causes of trauma-related mortality in this area [4].

TBI is characterized by primary damage to the brain resulting from mechanical forces applied to the head at the time of trauma as well as delayed damage triggered by different mechanisms that evolve over time [5–7]. TBI secondary injury includes a complex cascade of biochemical events involving oxidative stress, glutamate excitotoxicity, and neuroinflammation, leading to neuronal cell death [8]. Mitochondrial dysfunction at the neuronal/astrocytic level has been reported to be a key participant in neuroinflammation [9] and also in TBI pathophysiology [10, 11], leading to a marked reactive oxygen species (ROS) accumulation.

Oxidative stress, the imbalance between the level of ROS/reactive nitrogen species (RNS) and antioxidants, has been extensively investigated as one of the major contributors to the pathophysiology of secondary TBI damage. The most commonly occurring cellular free radical is superoxide (O_2_^•−^), which promotes the formation of other ROS/RNS leading to lipid peroxidation [12]. Mitochondria has been generally considered the main source of O_2_^•−^ following brain injury [13]; however in the last years NADPH oxidase (Nox) family members have emerged as major contributor to O_2_^•−^ generation. Several studies have demonstrated that Nox is upregulated after TBI [14–18] and pharmacological and genetic Nox inhibition has been shown to markedly attenuate TBI secondary injury [18, 19], suggesting Nox critical role in the onset and development of this pathology.

The following review summarizes current research on the damaging role of oxidative stress in TBI, focusing on NADPH oxidase as ROS generator enzymes.

## 2. Pathophysiology of Traumatic Brain Injury 

Traumatic brain injury (TBI) is a damage to the brain due to an external physical insult that can lead to loss of consciousness, impairment of cognitive and motor abilities, and disruption of behavioral and/or emotional functioning. These neurological deficits can be temporary or permanent and may lead to physical and psychosocial disability [20]. The outcome may vary from death to surviving with disabilities or even to complete recovery. The most common causes of TBI in adults are road traffic accidents, falls, violence, and armed conflicts [7].

The head trauma can be penetrating or closed according to the mechanism while the clinical severity is usually classified according to the Glasgow Coma Score (GCS) [21]. TBI patients are categorized into mild, moderate, and severe. A GCS score of 13–15 is conventionally associated with mild TBI, a score of 9–12 with moderate TBI, and a score of 8 or less with severe TBI [22].

TBI is characterized by primary and secondary damage. The primary damage is the direct expression of the mechanical forces applied to the head (impact, blast, and penetrating trauma) that cause localized and/or diffuse macroscopic brain lesions [23]. In particular in the case of severe TBI, focal and diffuse damage coexist: the localized damage includes focal contusions and hematomas, whereas diffuse damage includes brain swelling, microvascular damage and diffuse axonal injury (DAI). DAI is characterized by widespread damage to axons in the white matter [24, 25] that can be found up to 72% of moderate to severe TBI [26].

The severity of DAI can be classified in grade 1 or mild (changes diffusely distributed in the white matter but not in the* corpus callosum* or in the brainstem), grade 2 or moderate (with evidence of involvement of the* corpus callosum*), and grade 3 or severe (with additional aspects of lesion in the dorsolateral segments of the rostral brainstem) [27].

DAI might be considered a progressive process evolving from axonal damage to ultimate disconnection [28] and therefore, even if scarcely visible with conventional computed tomography (CT), can cause white matter disconnection that sustains cognitive, behavioral, and motor impairments and can heavily affect the short- and long-term outcome [29].

Moderate to severe TBI, as repeated mild TBI, can lead to long-term cognitive impairments and might be associated with increased risk of neurodegenerative diseases [30–32].

TBI also initiates a cascade of damage with variable extent and duration and with molecular mechanisms not yet completely understood. These processes take place for hours and days (or even weeks and months) after the brain trauma and may include hypotension, hypoxia, ischemia, excitotoxicity, and inflammation among others.

The secondary damage is non-mechanical, evolves over time [33], and comprises cytoskeletal damage and alteration of cell signaling pathways [34, 35]. A complex series of cellular and molecular changes play a fundamental role in these cascades and include blood-brain barrier (BBB) impairment [36–38], ionic imbalance [39], excitotoxicity [40], brain edema [41], neuroinflammation [9, 42, 43], and oxidative stress [44, 45].

In particular, the ischemic pattern observed in TBI impairs the capacity of neurons and glial cells to maintain membrane ionic equilibrium. As a consequence, depolarization occurs in neurons, resulting in activation of presynaptic voltage-dependent Ca^2+^ channels and in massive release of excitatory neurotransmitters like glutamate and aspartate into the extracellular space [46, 47]. The toxic level of excitatory amino acids activates postsynaptic NMDA (N-methyl-D-aspartate) and metabotropic receptors, which induce calcium overload of the postsynaptic neurons [48]. The final event in ischemic damage is always a massive intracellular Ca^2+^ accumulation [22] which leads to mitochondrial dysfunction and oxidative stress [49, 50]. Furthermore, excessive cytosolic calcium activates proteolytic enzymes and phospholipases that induce degradation of cytoskeleton and extracellular matrix proteins and enhances ROS production. [47]. Large lines of evidences demonstrate that ROS generation and oxidative stress contribute significantly to the pathophysiology of secondary injury after TBI [48, 51, 52].

## 3. Reactive Oxygen Species and Oxidative Stress

ROS, historically considered as purely harmful byproduct of metabolism causing cell damage, are now considered as important modulators of intracellular signaling pathways, since they can be intentionally generated in particular by the Nox family [53]. Accumulating evidence suggests that ROS are involved in several pathophysiological responses ranging from cell proliferation to cell death and that deregulated ROS signaling contributed to a multitude of human diseases, such as brain injury and neurodegenerative disease [54–59].

When cells, including neurons, are in a homeostatic balance, the availability of antioxidant enzymes (e.g., catalase, superoxide dismutase, glutathione peroxidase, glutathione reductase, and glutathione-S-transferase) and of scavenging molecules (e.g., glutathione, ascorbic acid, and tocopherols) approximately matches ROS level, allowing redox signaling [60].

Oxidative stress, on the contrary, represents the imbalance between ROS level and antioxidant defense and may arise from increased ROS formation or from deficiencies in antioxidant levels [61].

A variety of pathologies have been reported to be related to oxidative stress that causes irreversible oxidative modifications of proteins, lipids, and/or DNA, generating oxidative stress markers (e.g., carbonylated proteins, lipid peroxidation) and leading to cellular necrosis or apoptosis and consequently to tissue injury [53, 62]. Superoxide can directly or indirectly damage DNA through oxidation [63], directly inactivate cellular antioxidants enzymes [64], and activate proinflammatory nuclear factors [65]; therefore, it has been implicated in numerous pathological processes including acute and chronic diseases [66].

Other ROS/RNS that possess different redox characteristics and, thus, different physiological and pathophysiological effects can derive from superoxide. For example, colocalization of superoxide at sites of nitric oxide (NO) production can lead to the formation of peroxynitrite (ONOO^−^) and to oxidative damage due to peroxynitrite decomposition products that possess potent free radical features [62]. Moreover, superoxide is rapidly reduced, both spontaneously and enzymatically, to H_2_O_2_. Unlike superoxide, H_2_O_2_ is able to cross cellular membranes, through specific aquaporin channels [67], acting at sites distant from its source and modifying DNA and proteins [68]. Moreover, H_2_O_2_, in the presence of transition metals, can generate hydroxyl radical (OH^∙^) which is highly reactive and indiscriminately oxidizes nucleotides causing breaks and lesions of DNA and lipid peroxidation. The brain is highly susceptible to lipid peroxidation because of its elevated oxygen consumption and richness in polyunsaturated fatty acids [69] and iron [70]. Lipid peroxidation causes alterations in cell membrane fluidity, increases permeability of membranes, and decreases membrane activity, leading to cell injury.

The understanding of oxidative stress mechanisms and the development of antioxidant strategies are of primary interest to optimize brain injury treatment and may provide useful therapeutic strategies for brain injury inflammation and neurodegenerative diseases [56, 71–73].

## 4. Oxidative Stress in Traumatic Brain Injury

ROS generation has a profound impact on the onset of TBI secondary injury. The impaired blood flow following TBI triggers cerebral hypoxia or ischemia with the consequent reduction of oxygen and glucose supply to the brain. The transition from aerobic to anaerobic metabolism generates a state of acidosis which activates pH-dependent calcium channels [74]. The increased Ca^2+^ levels into neuronal cytoplasm lead to an increase in ROS/RNS production mainly due to the impairment of the mitochondrial electron transport chain and the activation of the calcium dependent proteases and phospholipases [74–76]. During blood flow restoration or reperfusion, enzymes involved in ROS production find enough oxygen to generate large quantities of ROS/RNS strongly contributing to oxidative stress in TBI [75]. The most common free radical generated almost immediately following TBI is superoxide [77, 78]. Within the injured nervous system, different possible sources contribute to the production of superoxide radical. Ca^2+^ induces activation of phospholipases and the downstream arachidonic acid cascade, xanthine oxidase activity, mitochondrial leak, enzymatic or autoxidation of biogenic amine neurotransmitters, oxidation of hemoglobin, and Nox family member activation.

At later time, TBI triggers a series of inflammatory processes that contribute to neuronal damage and failure of functional recovery. These processes are mediated by infiltrating inflammatory cells like activated microglia, neutrophils, and macrophages that produce multiple proinflammatory mediators, such as cytokines, chemokines, inducible NOS and cyclooxygenase-2 (COX-2), and can be additional sources of O_2_^•−^ [11, 79, 80].

In aqueous environments, like the cytoplasm, O_2_^•−^ exists in equilibrium with the hydroperoxyl radical (HO_2_^•^) which is more lipid soluble and a more powerful oxidizing agent [81]. However, under the acidic condition characteristic of TBI, there is a shift of the equilibrium in favor of HO_2_^•^ increasing lipid peroxidation. Lipid peroxidation can induce brain tissue damage by different mechanisms: impairing mitochondrial membrane lipid structure leading to mitochondrial dysfunction [82, 83]; enhancing the accumulation of 4-HNE that inhibits astrocytic glutamate transporters [84, 85], potentially increasing the neurotoxicity mediated by glutamate; compromising Ca^2+^ homeostasis by damaging the Ca^2+^-ATPase in the cell membrane [86]; and mobilizing Ca^2+^ from intracellular stores like the endoplasmic reticulum [87].

Another important player in post-TBI pathophysiology scenario is peroxynitrite, produced by coupling NO with superoxide. The damaging role of ONOO^−^ in TBI has been indirectly demonstrated by the neuroprotective effect of acute treatment of injured mice and rats with NOS inhibitors [88, 89] and by the use of the peroxynitrite derived free radicals scavenger, tempol, that ameliorated the accumulation of nitrotyrosine in injured brains and concomitantly improved neurological recovery in mice [90]. It has also been reported a significant upregulation of all the three NOS isoforms (endothelial, neuronal, and inducible) after TBI [91–93] with a consequent increase of NO level.

Red blood cell lysis, due to mechanical trauma, is another important source of oxidative stress in TBI. The main consequence of red blood cells lysis is the release of free hemoglobin [94] whose oxidation to oxyhemoglobin and methemoglobin contributes to ROS generation [95–98]. Moreover, hemoglobin degradation by either H_2_O_2_ or lipid hydroperoxides (LOOH) gives rise to the release of iron anions which further contributes to the formation of ROS/RNS [75]. Iron is tightly regulated in the brain under physiological conditions but after traumatic injury iron homeostasis is disrupted by acidosis that increases iron solubility and mediates its delocalization from an inactive to an active redox state [99, 100]. In conclusion, in TBI the many different ROS sources synergistically contribute to the onset of an extensive and profound condition of oxidative stress.

## 5. NADPH Oxidase Enzymes

ROS have been long thought to be only a harmful by-product of the electron transport chain in mitochondria or in enzymatic processes such as nitric oxide synthase (NOS), cytochrome p450, cyclooxygenase, xanthine oxidase, and lipoxygenase. Whereas the enzymatic processes listed above produce ROS as a side-reaction of normal enzymatic function, the accidental production of ROS is not the only modality of ROS generation. In fact, there are a family of membrane enzymes that reduce molecular oxygen to form ROS as their unique enzymatic function: these enzymes are called NADPH oxidases (Nox) since they use NADPH as a source of electrons to reduce molecular oxygen.

The first enzyme to be discovered that “intentionally” generates ROS in mammalian cells is the Nox expressed in the phagocytes. The phagocytic NADPH oxidase (now known as Nox2) is a membrane enzyme that produces large amounts of ROS in a “respiratory burst” characterized by consumption of O_2_ and production of superoxide and hydrogen peroxide that, in turn, can lead to the production of more reactive species such as peroxynitrite and hypochlorous acid (HOCl) [101].

Nox2 is a membrane enzymatic complex; the catalytic subunit (known as gp91*phox*) is an integral protein containing both flavin adenine nucleotide (FAD) and a heme group. Other components of the functional complex are the membrane protein p22phox which functions as a docking site for the cytosolic regulator proteins p40phox, p47phox, and p67phox and the small GTPase Rac. When assembled and activated, Nox2 is able to transport electrons from cytosolic NADPH to reduce molecular oxygen to form superoxide to the other side of the membrane.

Nox2 is expressed in neutrophils and other phagocytic cells mediating host defence against microorganisms; in these white cells Nox2 produces high levels of ROS in order to kill phagocytised microbes. The importance of Nox2 in the host defence is highlighted by the fact that mutations in the NADPH oxidase subunit genes can lead to chronic granulomatous diseases (CGD) [102].

Several homologs of gp91*phox* (Nox2 catalytic subunit) have been identified in non-phagocytic cells; now, the human Nox family consists of seven different isoforms (Nox1, Nox2, Nox3, Nox4, Nox5, Duox1, and Duox2) [101]. In addition, new regulatory proteins have been discovered, NOXO1 (NOX organizer 1) is homolog of p47*phox*, and NOXA1 (NOX activator 1) is homolog of p67*phox*. Owing to their structure and regulation Nox enzymes are categorized into two groups: isoforms that require p22*phox* (Nox1, Nox2, Nox3, and Nox4) and enzymes regulated by calcium through a calcium-binding domain (Nox5, Duox1, and Duox2) [103].

Nox isoforms are distributed in a variety of tissues and cells but, often, high expression of a certain isoform is only found in specific organs or cells. For instance, Nox1 is highly expressed in colon, Nox4 in the kidney, Nox3 in the inner ear, and Duox2 in the thyroid [104]. Nox-derived ROS levels in nonphagocytic cells are typically much lower than in neutrophils since they are not generated to host defense, but as second messengers molecules in response to physiological* stimuli* such as endothelial growth factor, platelet-derived growth factor, angiotensin II, and insulin.

The most abundant isoforms expressed in the brain are Nox1, Nox2, Nox3, and Nox4. Several studies have investigated the expression of Nox isoforms in specific CNS regions, most of them are focused on Nox2 but data exist also for Nox1, Nox3, and Nox4 [105]. Although available studies do not provide a complete description of the CNS distribution of Nox enzymes, it appears that Nox isoforms are often coexpressed in various CNS regions and that Nox expression in a certain CNS regions appears to be inducible rather than constitutive. Expressions of NOX isoforms in specific CNS cell types have been investigated* in vitro* on primary cultures. Nox1, Nox2, and Nox4 are present in neurons, astrocytes, and microglia but, unfortunately, the relative amount of different Nox enzymes and their peculiar function in different brain cells are not sufficiently understood [105].

## 6. NADPH Oxidase in Traumatic Brain Injury

It has been widely demonstrated that NADPH oxidase plays a key role in central nervous system pathophysiology [17, 106, 107] and increasing lines of evidence suggest that NADPH oxidase is major producer of O_2_^•−^ and has a crucial role in the development of secondary injury after TBI [18, 19, 108, 109].

Dohi et al. [110] were the first to evidence a direct involvement of NADPH oxidase in TBI injury. They demonstrated that gp91phox (also known as Nox2) is increased in the ipsilateral hemisphere after TBI and specifically in amoeboid-shaped microglial cells. Moreover, gp91phox^−/−^ mice exhibit reduced primary cortical damage and a lower ROS level after TBI. Several studies have indirectly investigated Nox involvement in TBI by the use of Nox inhibitors like apocynin [111–113] that acts through the inhibition of p47phox subunit translocation to catalytic subunit. Choi et al. [19] observed that intraperitoneal delivery of apocynin to rats before TBI decreased ROS production, BBB disruption, microglia activation, and exerted pronounced neuroprotection. The protective effect of Nox inhibition by apocynin was further investigated by Ferreira et al. [108] that evidenced that early treatment with apocynin reduced inflammatory and oxidative damage caused by moderate fluid percussion injury in mice (mLFPI). They also observed that apocynin did not show any protective effect on brain water content, suggesting that NADPH oxidase activity is not involved in the development of brain edema induced by TBI. On the contrary, other authors showed that NADPH oxidase inhibition reduces brain edema induced by cold brain injury and controlled cortical impact [18, 114]. One possible reason for these discrepancies could be found in the different apocynin doses used in the studies.

**Figure 1:**
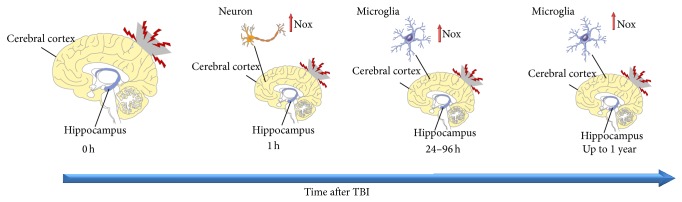
Time related changes of Nox expression after TBI.

Recent studies have shown a time-dependent change in Nox function following TBI (Figure 1). Zhang et al. [18] evidenced that Nox activity in the cerebral cortex and hippocampus rapidly increases following TBI with an early peak at 1 h, followed by a secondary peak at 24–96 h. In particular, they suggested that the first peak is of neuronal origin as demonstrated by a strong colocalization of Nox2 and O_2_^•−^ in neurons at 1 h after TBI; whereas the cellular source for the Nox and O_2_^•−^ elevation at 24–96 h appears to be activated microglia. These data were confirmed by a recent study by Lu et al. [15] that evaluated the temporal pattern of Nox2 activation in adult male mouse cerebral cortex following TBI. They observed a rapid and robust elevation of Nox2 expression in the cerebral cortex at 1 h, followed by a decrease to lower, but still elevated levels at 3–12 h after TBI. A second significant elevation was observed at 24 h after TBI with no significant difference in Nox2 expression at 72 h. These data have been challenged by Ansari et al. [14] that reported that both the Nox activity and O_2_^•−^ increased in a time-dependent fashion, with the maximum values at 24 h. The authors postulated that these discrepancies could be ascribed to the different animal model used and to the lack of inhibitors for other sources of O_2_^•−^ during the detection procedure used by Zhang et al. [18] and suggested that such an early peak is most probably associated with mitochondrial dysfunction that is known to occur within 30 min after TBI [16]. Song et al. demonstrated a significant Nox activation between 48 and 72 h after a diffuse brain injury [17] but they did not measure Nox activity before 48 h. Dohi et al. [110] showed that Nox is highly expressed in chronically activated microglia up to 4 months after TBI, and recently Loane et al. [115] extended this time period observing that Nox is upregulated in highly activated microglia surrounding the lesion site up to 1 y. Microglial Nox might cause neurotoxicity through two related mechanisms: activation of Nox leads to the production of extracellular ROS that are cytotoxic to neighboring neurons. Moreover microglial intracellular ROS produced by Nox are a key driver of self-propagating cycles of microglial-mediated neurodegeneration as Nox activation induces changes in microglia morphology and proinflammatory gene expression [116]. Given this dual effect of Nox activation on neurotoxicity, the role of Nox in increasing ROS level and the prevalence of Nox activation upon microglial activation suggested that microglial Nox could play a key role in neuronal death after TBI [117]. In the aged brain these effects are exacerbated as there is an exaggerated microglia activation in response to TBI with a Nox robust overexpression. In particular, in the injured cortex of aged mice a strong upregulation of the Nox subunits p22phox, and gp91phox has been observed [118].

The expression of Nox isoforms is dependent on cell type and injury status [119]. In particular, Nox2 is primarily expressed by microglia and neurons, Nox3 is primarily expressed by neurons, and Nox4 is expressed by all three cell types. Further, Nox2 is the most responsive to injury.

TBI is a well-known epigenetic risk factor for the development of later neurodegenerative diseases [120]. Nox2 activation in brain tissue and Nox2-induced oxidative stress have emerged as a critical factor in the pathogenesis of Alzheimer's disease [121, 122] and Parkinson's disease [116, 123].

Alzheimer's disease major hallmarks are the accumulation of *β*-amyloid and neurofibrillary tangles in the brain and the loss of neurons from the hippocampus and cerebral cortex [124]. It has been shown that TBI induces an accumulation of *β*-amyloid in the brain, which may explain the increased risk for cognitive decline and dementia in TBI patients [125, 126]. Interestingly, Nox inhibition by apocynin significantly attenuated the elevation of *β*-amyloid protein levels in the cortex following TBI, suggesting that Nox activation is involved in the induction of *β*-amyloid formation [18].

TBI was also found to induce overexpression of *α*-synuclein, the principal component of Lewy bodies, reported as a cause of Parkinson's disease [127, 128]. A recent study of Acosta et al. suggested *α*-synuclein as the pathological link between chronic effects of TBI and PD symptoms [129]. It has been shown that the knockdown of Nox in the substantia nigra largely attenuated the increase of *α*-synuclein in a paraquat-induced Parkinson's disease model, suggesting that Nox is involved in the mechanism responsible for generation of oxidative stress conditions implicated in increased *α*-synuclein expression and aggregation and dopaminergic neurodegeneration [130].

In summary, Nox upregulation occurs immediately after TBI and lasts for several days significantly contributing to oxidative stress damage and neuronal cell death. In particular, different lines of evidences suggest that Nox may be a causative factor in the onset of neurodegenerative disease related to TBI.

Although in terms of potential therapeutic strategies TBI treatment requires a deeper understanding of cerebral pathophysiology as well as of the neuroprotective responses toward therapeutic agents, targeting Nox isoforms could offer an intriguing hypothesis to decreasing/delaying the progression of temporary or permanent neurologic deficits that may result in lifelong impairment of physical, cognitive, and psychosocial functioning.

## 7. Conclusions

**Figure 2:**
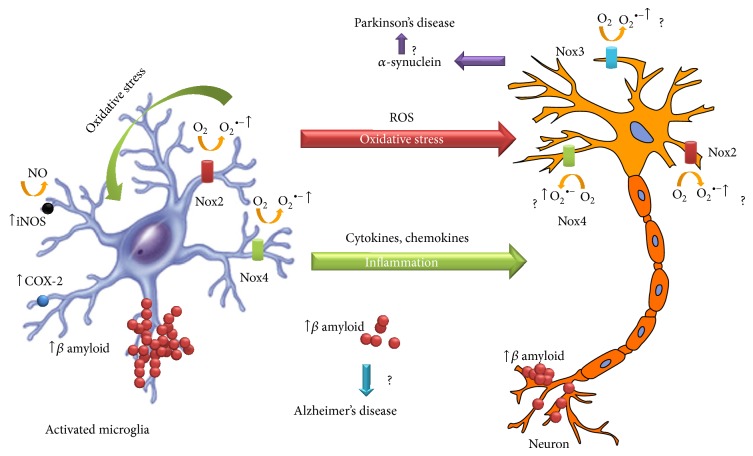
Schematic representation of the proposed mechanisms triggering cell damage after TBI.

Accumulating evidence suggests that Nox-derived ROS play a crucial role in TBI. Nox upregulation occurs immediately after TBI and lasts for several days significantly contributing to oxidative stress damage and neuronal cell death. ROS produced by Nox contribute to diseases by means of distinct mechanisms, such as oxidation of macromolecules and consequent modulation of redox signaling pathways (Figure 2). Despite the progress made in the understanding of oxidative stress involvement in the pathology of several neurodegenerative diseases, much remains to be learned about how to counteract neurological damage following TBI.

TBI is a well-known epigenetic risk factor for the development of later neurodegenerative diseases such as Parkinson and Alzheimer. Interestingly, Nox2-induced oxidative stress has emerged as a critical factor both in TBI secondary injury and in the pathogenesis of Alzheimer and Parkinson diseases suggesting that Nox2-generated ROS after TBI could be the cause of the increased neurodegenerative risks associated with TBI.

In summary, this review highlights the crucial role of Nox in TBI and suggests that selective and specific Nox inhibitor compounds could be useful for the development of novel therapeutic targets and strategies, allowing a fine correction of detrimental aspects of TBI.
